# Sulphur in Typhoid

**Published:** 1897-03

**Authors:** E. W. Berridge


### SULPHUR IN TYPHOID.

On May 14th, 1896, the patient inhaled the effluvium from a drain; felt ill ever since, and took to her bed on May 18th. (A very short period of incubation.) She was at first attended by an unqualified practitioner. As she became worse the patient's husband invested the quack with the "ancient and honorable order of the sack," and sent for me. One dose of *Sulphur*$^{dm}$ (F. C.) removed the following peculiar symptoms: Delusions at night, as if she did not know whether she was married or single, whether she was a woman or a man; also that her ears had been changed for some one else's ears, and that they seemed too large and sticking out; this confusion of mind prevented sleep. *Arsenicum*$^{cm}$ (F. C.) removed a feeling as if she was sinking through the bed. *Pulsatilla*$^{cm}$ (F. C.) was also given, chiefly for the symptoms of agreeable odors (such as champagne); this symptom it removed, with general improvement. She made a good recovery. E. W. BERRIDGE, M. D.

